# Development of MicroRNA Therapeutics for Hepatocellular Carcinoma

**DOI:** 10.3390/diagnostics3010170

**Published:** 2013-03-15

**Authors:** Rajagopal N. Aravalli

**Affiliations:** Department of Radiology, University of Minnesota Medical School, MMC 292 Mayo Memorial Building, 420 Delaware Street S.E., Minneapolis, MN 55455, USA; E-Mail: arava001@umn.edu; Tel.: +1-612-626-8174; Fax: +1-612-626-5580

**Keywords:** HCC, microRNA, replacement therapy, liver cancer, animal model

## Abstract

Hepatocellular carcinoma (HCC) is the most common form of liver cancer and is the third leading cause of cancer-related deaths worldwide. Treatment options for HCC are very limited, as it is often diagnosed at a late stage. Recent studies have demonstrated that microRNAs (miRNAs), a class of non-coding RNAs, are aberrantly expressed in HCC. Some of these were shown to be functionally involved in carcinogenesis and tumor progression, suggesting that miRNAs can serve as novel molecular targets for HCC therapy. Several promising studies have recently demonstrated the therapeutic potential of miRNAs in animal models and in reducing the viral load in hepatitis C patients. In this review, these advances and strategies for modulating miRNAs for *in vivo* therapeutic delivery and replacement therapy are discussed.

## 1. Introduction

Human liver cancer can be classified either as hepatoblastoma, hepatocellular carcinoma (HCC), angiosarcoma or cholangiocarcinoma. Among these cancer types, HCC is the most common type that accounts for approximately 70–80% of all primary liver cancer cases [1]. HCC normally develops as a consequence of underlying liver disease and is often associated with cirrhosis. A variety of risk factors contribute to the initiation of HCC [2]. Surgical resection and whole liver transplantation are the current best options for treatment. Recurrence is a common occurrence in patients who have had a resection, and the survival rate is 30–40% at five years post-surgery [3]. 

Hepatocarcinogenesis is a multistep process involving a number of factors, such as mutations that cause genetic alterations, aberrant expression of cellular proteins, inhibition of tumor suppressors, overexpression of oncogenes and molecules that regulate these events, including microRNAs (miRNAs) and various cellular proteins [2]. Initial studies on HCC pathogenesis have identified a number of critical signaling pathways, as well as the mutations that activate oncogenes (*β-catenin*, *Axin1*, *PI-3-kinase*, *K-ras*) and inactivate tumor suppressors (*p53*, *Rb1*, *CDKN2A*, *IGF2R*, *PTEN*) [2,4]. Subsequent efforts to identify potential prognostic and diagnostic biomarkers have resulted in the development of the first FDA approved drug for HCC: sorafenib, a multi-kinase inhibitor [5]. Sorafenib has been successful, because it targets proteins of multiple signaling pathways simultaneously—VEGFR2, VEGFR3, PDGFR, Flt-3, c-kit and the Raf/MEK/ERK pathway—to inhibit tumor growth and to induce apoptosis of tumor cells [6,7]. The success of sorafenib has resulted in the development of a number of novel therapeutic compounds. These include antibodies, small peptides, heparin-degrading endosulfatases and oncolytic viral agents [2]. 

Another emerging area of HCC therapeutics is based on miRNAs. miRNAs are small (~21–23 nucleotides long), non-coding RNAs that regulate post-transcriptional gene expression of their target genes either by inducing translational repression via their binding to partially complementary sequences or by directing mRNA degradation through their binding to perfectly complementary sequences in the 3*'* untranslated region (UTR) of messenger RNAs (mRNAs) [8]. Each mature miRNA potentially controls many gene targets, and each mRNA is regulated by multiple miRNAs. To date, more than 17,000 distinct mature miRNA sequences have been identified from over 140 species [9]. A number of recent studies have documented the involvement of miRNAs in HCC in tumor progression and metastasis [10,11,12,13,14,15]. In humans, more than 50% of miRNA genes are located at fragile sites or in cancer-associated genomic regions that are frequently involved in chromosomal abnormalities, such as loss of heterozygosity, amplification and breakpoints [2], indicating that they might be useful as novel diagnostic and/or prognostic markers and could constitute potential molecular targets in cancers. 

miRNAs are transcribed by RNA polymerase II to form primary transcripts (pri-miRNAs), capped with 7-methylguanosine and polyadenylated. These pri-miRNAs are processed in the nucleus by the RNase III enzyme Drosha and its cofactor, Pasha/DGCR8, into ~60–70 nucleotide precursors (pre-miRNAs), which form an imperfect stem-loop structure and are exported into the cytoplasm by the RAN GTP-dependent transporter, Exportin 5. RNase III enzyme Dicer then processes pre-miRNAs into mature miRNAs, which are subsequently loaded onto the RNA-induced silencing complex (RISC). The miRNA/RISC complex then binds to the 3*'*-UTR of target mRNAs and downregulates their expression [16,17]. Thus, miRNAs modulate various cellular signaling pathways involved in cell growth, proliferation, motility and survival. miRNAs are also subjected to regulation by epigenetic mechanisms, and mutations in their promoter and coding regions were shown to contribute to tumorigenesis [18,19,20,21,22]. 

## 2. Involvement of MicroRNAs in HCC

During the past decade, it has been well established that specific miRNAs modulate various cellular processes in the liver and that their aberrant expression correlates with the severity and poor prognosis of HCC [23,24,25,26,27]. For example, in one study, the expression of miR-199a, miR-92, miR-106a, miR-222, miR-17-5p, miR-18 and miR-20 correlated with the degree of tumor differentiation, suggesting the involvement of these miRNAs in disease progression [23], while in another study, miRNAs associated with viral infection and the stage of liver disease were identified by comparing miRNA expression profiles of patients with hepatitis B virus (HBV)-related HCC with those from HCV-related HCC [1,25]. Further analysis of HBV-associated miRNAs revealed that their targets included genes involved in pathways related to cell death, DNA damage, recombination and signal transduction, and the targets for HCV-associated miRNAs included genes involved in immune response, antigen presentation, cell cycle, proteasome and lipid metabolism [25]. This finding demonstrated that HBV and HCV induce different sets of miRNAs during infection. 

miRNA-122, an abundant liver-specific miRNA that modulates hepatic lipid metabolism [28], is often downregulated in human HCC [29,30]. Loss of its expression correlates with loss of mitochondrial metabolic function and is detrimental to sustaining critical liver function, thereby contributing to the morbidity and mortality of liver cancer patients [31]. In the HBV-related HCC cell line, HepG2.2.15, miR-122 inhibited viral replication by targeting NDRG3, a member of the *N-myc* downstream-regulated gene family [32], suggesting that both miRNA-122 and NDRG3 are viable therapeutic targets for HBV-related HCC. During HCV infection, however, miR-122 binds directly to two sites in the 5*'* non-coding region of HCV genome and positively regulates the viral life cycle [29]. Thus, inhibition of miR-122 presents an attractive treatment option for HCV infection. 

In another study, examination of miRNAs in HCC with cirrhotic background revealed that members of the let-7 family, miR-221 and miR-145 were downregulated [33]. In these tissues and in HCC cell lines, miR-122 was also downregulated, and its target gene product, cyclin G1, was highly expressed, promoting the growth of cancer cells. However, when miR-122 expression was restored, it significantly reduced *in vitro* migration, invasion and anchorage-independent growth of Mhalavu and SK-HEP-1 cells, as well as *in vivo* tumorigenesis, angiogenesis and intrahepatic metastasis in an orthotopic liver cancer model by inhibiting ADAM17, a protein involved in metastasis [34]. Interestingly, miR-122 also inhibited tumorigenic properties of HCC and sensitized tumor cells to sorafenib [32]. Collectively, these studies demonstrate that miR-122 can function both as a positive and negative regulator in liver cancer. Other studies have shown that miRNAs associated with cell cycle inhibition (miR-34a, miR-101, miR-199-a-5p and miR-223) were downregulated in HCC [22,35,36,37,38,39,40], and those involved in cell proliferation and inhibition of apoptosis (miR-17-92 polycistron, miR-21, miR-96, miR-221 and miR-224) were upregulated [41,42,43,44]. Furthermore, miRNA-221 is associated with tumor multifocality [45]. 

As demonstrated in these examples (Table 1), aberrant miRNA expression leads to the dysregulation of critical cellular mechanisms and activation of tumorigenic pathways involved in tumor differentiation, diagnosis, staging, progression, prognosis and response to therapy [28,29,30,31,32,33,34,35,36,37]. 

**Table 1:** Differentially expressed miRNAs in liver tissues and hepatocellular carcinoma (HCC) cell lines.

Sample type	Method	miRNAs *	Cellular Target/Mechanism	Reference
Tumor tissues	Microarray, qPCR	miR-199a, miR-92, miR-106a, miR-222, miR-17-5p, miR-18, miR-20	Tumor progression	[23]
Tumor tissues, rat model of hepatoma	Microarray, Northern blot	*miR-122*, *let-7a*, miR-21, miR-23, miR-130, miR-190, miR-17-92 family	Tumorigenesis	[30]
Tumor tissue	Microarray	*miR-122*	Loss of mitochondrial metabolism	[31]
HCC cell lines	qPCR	*miR-122*	NDRG3	[32]
Tumor tissues, HCC cell lines	Microarray, qPCR Northern blot	*miR-122* *let-7 family, miR-145*	Cyclin G1	[33]
HCC cell lines	Western blot, Soft agar assay	*miR-122*	ADAM17, migration, invasion, anchorage-dependence, angiogenesis, metastasis	[34]
Tumor tissues, HCC cell lines	qPCR, Western blot	*miR-34a*	c-Met, apoptosis, cell cycle arrest, Senescence	[36]
Tumor tissues, HCC cell lines	Microarray, Northern blot, Western blot	*miR-101*	Mcl-1, apoptosis, tumor suppression	[39]
Tumor tissues	Microarray, qPCR	*miR-199-a-5p, miR-223*	Cell cycle inhibition	[22,35,36,37,38,40]
Human and Woodchuck HCC cell lines	qPCR, Northern blot	miR-17-92, miR-21	Cell proliferation, apoptosis	[41]
Tumor tissues	qPCR, Northern blot	miR-221	CDKN1C/p57, CDKN1B/p27	[42]
Tumor tissues	Microarray, qPCR, Northern blot	miR-21	PTEN	[43]
Tumor tissues	Microarray	miR-224	Apoptosis inhibitor-5	[44]

* Downregulated miRNAs were shown in italics.

## 3. Strategies for the Modulation and Delivery of miRNAs

The application of miRNAs in cancer therapeutics normally involves one of the two strategies: (1) gain of function to inhibit tumor-inducing miRNAs (also termed as “oncomiRs”) using miRNA antagonists, such as locked nucleic acids (LNA), antagomiRs and antimiRs; and (2) miRNA replacement by re-introducing miRNAs with tumor suppressor functions to restore the loss of function [46]. While miRNA antagonists are oligonucleotides containing the complementary sequences of endogenous miRNAs, miRNAs used for replacement therapy can either be oligonucleotides or those expressed from transfected/transduced vectors. Endogenous miRNAs can be modulated by transient or stable transfection or by viral delivery of a small RNA molecule that can either be a pri-miRNA transgene, a pre-miRNA, a mature miRNA, a miRNA/miRNA*, a small interfering RNA (siRNA) or a short hairpin RNA (shRNA) [17]. The primary challenge for gene therapy is to develop a method that can deliver a therapeutic miRNA to target cells where proper gene expression can be achieved. An ideal delivery method should: (1) allow efficient entry of miRNA and its carrier into the target cell; (2) protect the RNA against degradation by nucleases in intercellular matrices; and (3) be nontoxic. Exogenous RNA (“miRNA modulator”) thus expressed with an appropriate delivery method can regulate the activity of miRNAs to alleviate tumorigenicity. 

To function effectively *in vivo*, however, miRNA modulators must cross the membranes of tumor cells first and then bind their targets stably in the cytoplasm. In general, viruses are able to mediate gene transfer with high efficiencies, as they can infect every cell in a tumor tissue and may induce long-term expression of miRNAs in the infected cell. They can also be manipulated to alter their tropism so that they can infect the given cell type by binding to the cell surface receptor of choice. Therefore, viral vectors are the preferred choice for therapeutic studies of human diseases. However, acute immune responses, immunogenicity and insertion mutagenesis uncovered in gene therapy clinical trials have raised serious safety concerns on therapeutic application of commonly used viral vectors [47]. 

When compared with viral methods, nonviral delivery approaches are less efficient. These methods can be classified either as physical or chemical approaches. Physical methods employ a physical force that permeates the cell membrane and facilitates intracellular gene transfer, such as needle injection, electroporation, gene gun, ultrasound and hydrodynamic delivery; and chemical approaches use synthetic or naturally occurring compounds as carriers to deliver the transgene into cells [47]. These methods have encountered serious obstacles in recent years, because naked RNAs are highly unstable in biofluids and are degraded rapidly by cellular nucleases [11,32,33]. To overcome these challenges, efforts are being made to stabilize the miRNA by chemical modification, conjugation and encapsulation; and to improve its delivery to the target tissues and tumor cells *in vivo*. Some of the promising methods successfully employed in cancer research (Table 2) are discussed below.

**Table 2:** Successful miRNA-based therapeutic studies reported for various types of cancer.

Therapeutic strategy	miRNA modulator/overexpression	Cancer	Delivery method ( *in vitro*/*in vivo*)	Reference
miRNA inhibition	LNA-anti-miR-19	Breast	Transfection	[48]
	LNA-miR-135b	Lymphoma	Lentivirus	[49]
	Anti-miR-21/anti-miR-155/anti-miR-17-5p/anti-miR-1/anti-miR-133AMO	Breast	Transfection	[50]
	Anti-miR-221-AMO	Liver	i.v. injection	[51]
	AntagomiR-17-5p	Neuroblastoma	Lentivirus	[52]
	AntagomiR-221/222	Prostate	s.c. injection	[53]
	Anti-oncomiRs miR-143/miR-145/liposome complexes	Colorectal	i.v. injection	[54]
	miR-145/PU-PEI complexes	Glioblastoma	s.c. injection	[55]
	LNA-miR-145/PU-PEI	Lung adenocarcinoma	s.c. injection	[56]
	MUC1 aptamer-miR-29b	Ovarian	Transfection	[57]
	Nucelolin-miR-122 aptamer	Brain	Transfection	[58]
miRNA replacement therapy	scFV antibody coupled to miR-34a nanoparticles	Lung	i.v. injection	[59]
	miR-34a mimic	Lung	s.c. injection	[60]
	miR-33a/PEI and miR-145/PEI complexes	Colon	i.p./i.v. injection	[61]
	miR-34a mimic	Colon	Transfection	[62]
	miR-34a and let-7 mimics miR-26a	Lung	xenografts	[63]
	miR-26a	Liver	AAV vector	[64]

AAV, adeno-associated viruses.

### 3.1. Stabilizing miRNA Inhibitors by Chemical Modifications

The base-pair interaction between miRNAs and their target mRNAs is essential for the function of miRNAs. Therefore, to silence miRNAs, nucleic acids that were antisense to the target miRNA have been developed. These antisense oligodeoxyribonucleotides (AMOs) specifically and stoichiometrically bind to their targets and silence them efficiently and irreversibly [50]. The AMO approach has recently been used successfully for miRNA therapy of human diseases [65,66]. By modifying this approach and by engineering various antisense miRNAs into a one single unit, Lu *et al*. showed that multiple target miRNAs can be silenced simultaneously in cell culture studies with the breast cancer cell line MCF-7 [50]. Despite this success, dose-response actions and *in vivo* stability of AMOs is largely unknown. Further studies are needed to further evaluate their potential in cancer therapy.

One of the first modifications to be carried out to enhance the stability of small RNAs was the substitution of 2*'*-OH in the ribose ring with 2*'*-*O*-methyl or 2*'*-fluorin. In *in vivo* experiments with rodents, antisense miRNA inhibitors carrying this substitution displayed enhanced stability and had a higher binding affinity to the miRNA target [67,68]. The use of phosphorothioate bond, that replaces the oxygen atom in the phosphodiester linkage with a sulfur atom, also improved stability of these small RNA to nuclease degradation [69]. AntagomirRs against miR-17-5p and miR-221/miR-222 containing the 2-*O*-methyl modification and terminal phosphothioates, together with a 3*'*-conjugated cholesterol group, significantly reduced tumor growth when administered subcutaneously in mouse models of neuroblastoma and prostate cancer, respectively [52,53]. This approach has since faced setbacks because of the requirement for high doses of antagomiRs [70]. 

The most commonly used miRNA inhibitors in cancer studies are the locked nucleic acid (LNA) antisense oligonucleotides [71]. LNAs are conformational RNA analogs that bind complementary RNA with extremely high affinity making them ideal candidates for cancer detection and analysis of cancer diagnostics. LNA-antimiRs have locked ribose rings that connect 2*'*-*O* and 4*'*-*C* atoms through methylene bridges, thus conferring unprecedented specificity, due to a significant increase in the RNA:RNA melting temperature [71]. The advantage of LNA-antimiRs is that they can be used in low doses, as they are relatively more resistant to nuclease digestion. Successful use of the LNA-based approach was also shown by high affinity inhibition of miR-91a and miR-21 in animal models of breast cancer [72,48].

Double-stranded RNA molecules containing the same sequence as the depleted, naturally occurring miRNA are commonly used as “miRNA mimics” for replacement therapy. They are expected to target the same set of mRNAs that is also regulated by the natural miRNAs [46]. To protect these dsRNAs from nuclease digestion and to avert immune responses, they were modified with the addition of inverted bases and alkyl or biotin groups [70,73]. While designing these dsRNAs, it is important to preserve target specificity and to ensure miRISC loading [69]. In a mouse model of human colorectal cancer, miR-143 duplex with the modified passenger strand and 3*'*-overhangs displayed greater activity and stability to nucleases in a mouse xenograft model of human colorectal cancer [54]. This chemically modified synthetic miR-143 also showed a significant tumor suppressive effect than the endogenous miR-143 [54].

More recently, a novel class of small molecule inhibitors of miRNAs have been developed [74]. By screening over 1,000 compounds at the 10 μM concentration, Gumireddy *et al*. identified two diazobenzene compounds that can inhibit miR-21 in three tumor cell lines [74]. It would be interesting to see if any of these could inhibit miR-21 *in vivo*.

### 3.2. Development of Carriers for Nonviral miRNA Delivery

Liposomes are commonly used to deliver DNA and RNA into the cells as they have higher half-lives in systemic circulation than naked oligonucleotides. Moreover, they protect oligonucleotides from degradation and nuclease digestion. In one study, neutral liposomes carrying synthetic miR-34a and let-7 mimics were able to reach the lung tissues when administered systemically in mice and inhibit tumor growth [60,63]. Dosage issues and *in vivo* stabilities of liposomes have resulted in the search for suitable alternatives. Polyethanolimine (PEI)-based polymers were developed as an alternative to liposomes. They are biodegradable and are capable of delivering miRNA mimics to the target tissues [68]. When administered in mouse models of colon cancer [61], glioblastoma [55] and adenocarcinoma of the lung [56], PEI-polymers carrying miR-34 and miR-145 mimics strongly inhibited tumor formation. 

Nanoparticles are popular delivery vehicles for human studies both *in vivo* and *in vitro*. Even though the nanoparticles made with type I collagen were frequently used to deliver siRNAs into cells, N- and C-linked telopeptides of collagen were found to elicit immune responses, thereby hindering the delivery of RNAs into cells [68]. To overcome these adverse effects, nanoparticles carrying miRNA inhibitors were made with atelocollagen, a type I biocompatible collagen [75]. These atelocollagen nanoparticles carrying miR-34a and LNA-miR-135b were shown to effectively inhibit colon cancer and lymphoma in mice, respectively [62,49]. More recently, nanoparticles carrying miR-143b were shown to inhibit tumors induced by human osteosarcoma cells [76] and orthotopic inoculation of nanoparticles with miR-516a-3p inhibited peritoneal dissemination of gastric cancer in mice [77]. These findings demonstrate that atelocollagen-mediated delivery of miRNAs would be an effective strategy for cancer treatment. 

Another approach for the delivery of miRNA inhibitors is the use RNA ligands, termed as “RNA aptamers”. They target specific cell surface receptors to facilitate the entry of the miRNA modulators that are coupled to them. One such chimera Chi-29b composed of a mucin 1 (MUC1) aptamer (that targets tumor cell surface MUC1 protein) coupled to miR-29b was recently shown to inhibit the growth of epithelial ovarian carcinoma cells *in vitro* by inducing apoptosis in a dose-dependent manner [57]. In a similar study, MUC1 aptamer coupled to let-7i miRNA inhibited the proliferation of these cells and sensitized them to paclitaxel treatment [78]. These findings suggest that RNA aptamers are useful vehicles for the delivery of miRNAs into tumor cells. However, their therapeutic utility *in vivo* is yet to be realized. 

Fluorescent DNA probes termed “molecular beacons” are being used to detect mature miRNAs in real-time imaging [79]. Kim *et al.* developed a cancer-targeting theranostics probe in a single system using the AS1411 aptamer and miRNA-221 molecular beacon (miR-221 MB)-conjugated magnetic fluorescence (MF) nanoparticle (MFAS miR-221 MB) to simultaneously target to cancer tissue, image intracellularly expressed miRNA-221 and to inhibit miR-221 in various brain cancer cell lines [58]. This novel method was sensitive enough to detect the miR-221 biogenesis in real-time and to produce antitumor therapeutic effects by inhibiting the miRNA function. Additional studies are needed to extend this simultaneous miRNA detection and inhibition approach to other cancers.

A more recent strategy to target cell surface receptors for miRNA delivery is based on antibodies. In this approach, single-chain variable fragment (scFv) antibodies with variable regions of immunoglobulin heavy and light chains were coupled to a short linker peptide of 10–25 amino acids and encapsulated into nanoparticles [68,80]. It has been established in melanoma studies that systemic delivery of scFv antibodies coupled to miR-34a mimics were able to reduce tumors without causing any adverse side-effects [59], thus demonstrating their potential use in cancer treatment. Synthetic RNA-based regulatory systems that integrate sensing and gene-regulatory functions, where the former are encoded in RNA aptamer sequences that recognize small molecule ligands, have also been developed [57]. Such integrated ligand-responsive RNA-based control systems offer several advantages over more traditional protein-based regulatory systems in avoiding the potential immunogenicity of heterologous protein components and providing a more tunable, compact control system [57]. 

### 3.3. Modulating Virus-Mediated Delivery of miRNAs

Viral vectors commonly used in human gene therapy are modified, noninfectious adenoviruses, adeno-associated viruses (AAV), herpes simplex viruses (HSV) and lentiviruses. Each of these can encode miRNA mimics and express them in infected cells. Since viral vectors can be engineered to alter their tropism for transduction into any cell type, they are the most preferred vectors for gene therapy. Recombinant adenoviral (Ad) and AAV vectors were commonly used in such studies, because they do not integrate into the host genome, but replicate as episomes in the nucleus and stably express miRNAs. Both Ad and AAV vectors are ideal for transient expression of transgenes in tumor cells or in cells that divide slowly [81]. Moreover, these vectors are of greater relevance, because random integration of lentiviral vectors may result in the activation of oncogenes and induce tumorigenesis. It was shown recently that AAV vectors transduce hepatocytes efficiently *in vivo* when injected through the intra-portal or intra-hepatic artery of healthy and cirrhotic rat livers, suggesting that they are convenient for transgene expression in the liver [82]. A major drawback of viral vectors, in general, is that they elicit strong immune responses in transduced cells, which reduces gene transfer and limits re-administration. Therefore, chemical or physical modification of Ad vectors with polymers has been used as a strategy for cancer therapy. Lately, to endow targeting moiety to polymer-coated Ad vectors, a diversity of ligands, such as tumor-homing peptides, growth factors or antibodies, have also been introduced to avoid unwanted transduction and to enhance therapeutic efficacy [83]. In addition, efforts are being made to encapsulate Ad vectors into poly (lactide-co-glycolide) (PLG) microspheres prior to administration [84]. When compared to free Ad vectors, PLG-encapsulated Ad vectors are known to have a significantly higher transduction efficiency rate in both E1 complementing and non-complementing cells [84]. When Ad was enveloped by cationic lipids, significantly high levels of viral uptake was observed in cultured HepG2 cells [85]. When administered in mice, these artificial envelopes can significantly alter the interactions with blood components and divert viral particles from their natural liver tropism, resulting in reduced hepatotoxicity [85]. Another added advantage of encapsulated viral vectors is that their level of transduction can be controlled by varying the quantity of encapsulated viral particles in the microspheres and the amount of microspheres administered.

AAV vectors, on the other hand, are single stranded adenoviruses devoid of critical genes involved in viral infection, making them relatively safe to administer in gene therapy trials. However, because of the difficulties in the construction of AAV vectors, self-complementary AAVs (scAAVs) that contain inverted repeats of its genome were developed. scAAVs can fold into dsDNA without the need for additional DNA synthesis or base-pairing between multiple vector sequences [68]. Successful therapeutic delivery of miR-26a-expressing AAV vector has recently been reported in a murine liver cancer model [64]. 

Efforts to knock-down miRNAs by using the classical RNA interference (RNAi) techniques were tested for inhibiting miRNAs. However, these approaches have largely been unsuccessful. Therefore, to induce the knockdown of miRNAs, genetic “sponges” were developed. These “miRNA sponges” are competitive inhibitors expressed from vectors containing multiple, tandem binding sites to a miRNA of interest [86]. When transiently transfected into cultured cells, miRNAs expressed from a strong promoter on these sponges will compete with endogenous miRNAs and sequester mature miRNAs away from their natural targets. This approach has been successfully used recently to knockdown miR-223 using a lentiviral vectors [87].

All these diverse methods of miRNA delivery and encapsulation would allow us to perform both loss-of-function and gain-of-function assays and to modulate the expression of miRNAs *in vivo*, thereby facilitating the application of miRNAs for cancer therapy.

## 4. Strategies for the Development of miRNA Therapeutics for HCC

The field of miRNA therapeutics for HCC is still in its infancy. So far, only a handful of successful outcomes have been reported. By using the above approaches that were successful with other cancers, experiments can be designed to suppress oncomiR expression and to restore the expression of downregulated miRNAs in tumor tissues of the liver. Some of the promising candidates for these miRNA therapeutics are discussed below.

### 4.1. Inhibition of OncomiRs

OncomiRs promote tumor growth and proliferation by blocking the activities of cellular tumor suppressors, cell cycle regulators and pro-apoptotic genes. For instance, miR-224 increases tumor cell progression by inhibiting the expression of apoptosis inhibitor-5 [18,44], and miR-221 promotes cell proliferation by controlling the cell cycle inhibitors (CDKI) CDKN1C/p57 and CDKN1B/p27 [42]. miR-519d, on the other hand, contributes to hepatocarcinogenesis by targeting CDKNA/p21, PTEN, AKT3 and TIMP2 [88]. Intratumoral administration of miR-143 showed that high levels of miR-143 can significantly promote HCC metastasis in an athymic nude mouse model by repressing the expression of fibronectin type III domain containing 3B (FNDC3B), which regulates cell motility [89]. miR-222, a frequently overexpressed miRNA in HCC, also regulates cell motility by enhancing Akt signaling [90], whereas miR-423 directly binds to 3*'*-UTR of p21Cip1/Waf1 to suppress its expression and to promote cell cycle progression and tumorigenesis [91].

While miR-146a increases the resistance of HCC cells from the cytotoxic effects of interferon-α by inhibiting the expression of Smad4 [92], upregulation of hepatic transforming growth factor (TGF)-β and its downstream mediators, Smads 2, 3 and 4, was found to be correlated with an increased expression of miR-181 in the livers of mice fed with a choline-deficient L-amino acid-defined (CDAA) diet [93]. Depletion of miR-181b inhibited tumor growth in nude mice, whereas its expressed enhanced resistance of HCC cells to the anticancer drug, doxorubicin [93]. miR-17-5p upregulates the migration and proliferation of HCC cells by activating the p38 mitogen-activated protein kinase MAPK pathway and increasing the phosphorylation of heat shock protein 27 [94], suggesting its potential application in HCC therapy. The miR-34 family members are direct transcriptional targets of tumor suppressor, p53, and loss of miR-34 function can impair p53-mediated cell cycle arrest and apoptosis [19].

miR-210, which is often upregulated in HCC, promotes hypoxia-mediated tumor cell metastasis by targeting the vacuole membrane protein 1 [95]. Therefore, inhibition of miR-210 expression could lead to reduction in HCC metastasis and should facilitate the development of novel therapeutic strategy against hypoxic tumor cells. Similarly, miR-30d, a miRNA associated with intrahepatic metastasis of HCC, promotes tumor cell migration and invasion *in vitro* and intrahepatic and distal pulmonary metastasis *in vivo* by targeting the Galphai2 (GNAI2) protein [96]. The expression of miR-148a is elevated in HepG2 and Hep3B cells, where it promotes cell proliferation, cell cycle progression and cell migration. When anti-miR-148a was overexpressed from a lentivirus, it inhibited the activity of miR-148a, in addition to blocking the Akt signaling pathway, in these cells, suggesting that it could serve as an early diagnostic marker and/or therapeutic target [97]. Additionally, miR-1 and miR-499 inhibited invasion and migration of HepG2 cells by targeting the ets1 proto-oncogene, which causes degradation of extracellular matrix [98]. Another antagomiR, miR-219-5p, induced tumor suppressive effects in HCC cell lines by targeting glypican-3 and by causing cell cycle arrest at the G1 to S transition [99].

While successful strategies to inhibit oncomiRs for HCC therapy have not been reported so far, each of these miRNAs represent a strong candidate for therapeutic intervention towards achieving a gain of function with the use of miRNA antagonists, RNAi and small molecule inhibitors. A recent study has showed the effects of a modified AMO against miR-221 in reducing the number and size of HCC tumors in a miR-221 transgenic mouse model [51]. While this study is promising, further in-depth studies on candidate miRNAs will allow us to ascertain their potential as therapeutic targets for HCC treatment.

### 4.2. miRNA Replacement Therapy

The first successful demonstration of miRNA replacement to restore the expression levels of a downregulated miRNA by delivery to the tumor was reported using a miR-26a-encoding AAV vector in a mouse model of HCC [64]. miR-26a is normally downregulated in HCC, and its overexpression in mouse livers results in the inhibition of cancer cell proliferation and induction of tumor-specific apoptosis [64]. More recently, another miRNA downregulated in HCC, miR-34a, has been successfully tested using this approach and by delivering it with NOV340 liposomes in an orthotopic model of HCC [100]. In both cases, significant tumor reduction, dramatic protection from disease progression without toxicity and prolonged survival of animals has been reported. While these reports were aimed to restore a loss of function, similar success has not yet been achieved with approaches to inhibit miRNA expression levels. All these studies are built on the regulation of multiple cellular pathways associated with human disease, which now appears to be a requirement for successful cancer therapy [101]. 

Along these lines, another downregulated miRNA in HCC, miR-375, was overexpressed in liver cancer cells. It decreased cell proliferation, clonogenicity, migration/invasion, induced G1 arrest and apoptosis [102] *in vitro*, indicating that it could be a good candidate for *in vivo* studies. Indeed, when cholesterol-conjugated 2*'*-*O*-methyl-modified miR-375 mimics (Chol-miR-375) were administered in nude mice, they were able to significantly suppress the growth of hepatoma xenografts [103]. 

In other promising studies, overexpression of miR-376a suppressed cell proliferation in HuH7 cells [101] and miR-138 induced cell cycle arrest by targeting cyclin D3 [103]. In an *in vitro* experiment with HCC cell lines transfected with miR-637 mimics or transduced with Lv-miR637 vectors, miR-637 suppressed tumor growth by negatively regulating the phosphorylation of STAT3 protein [104].

Downregulation of miR-29b correlates with rapid recurrence and poor survival of individuals with HCC. miR-29b dramatically suppressed the ability of HCC cells to promote capillary tube formation of endothelial cells and to invade extracellular matrix gel *in vitro* and in mouse xenografts where it regulated the expression of matrix metalloproteinase 2 [105]. Restoration of miR-29b expression resulted in the deregulation contributes to angiogenesis, invasion and metastasis of HCC [105], suggesting it can be used as a novel therapeutic target in anti-HCC therapy. 

Intratumoral injection of cholesterol-conjugated miR-99a mimics was recently demonstrated to significantly inhibit tumor growth and to reduce α-fetoprotein levels in HCC-bearing nude mice. In this study, miR-99a expression inversely correlated with protein levels of insulin-like growth factor 1 receptor (IGF-1R) and mammalian target of rapamycin (mTOR), inducing cell cycle arrest at the G1 phase [106]. This finding suggested a potential tumor suppressor role for miR-99a. Another miRNA often downregulated in HCC is miR-199a-3p. By transfecting pre-miR-199a-3p and anti-miR-199a-3p oligonucleotides, it was shown to target mammalian target of rapamycin (mTOR) and c-Met in HCC cells [107]. Restoring attenuated levels of miR-199a-3p in these cells led to cell cycle arrest at the G1 phase, reduced invasive capability, enhanced susceptibility to hypoxia and increased sensitivity to doxorubicin-induced apoptosis, suggesting that enhancement of miR-199a-3p levels may have a therapeutic benefits in HCC [107]. 

miR-122 binds to the 3*'*-UTR of Bcl-2 family member, Bcl-w, an anti-apoptotic protein, to induce cell death in HepG2 and Hep3B cells [108]. When expressed from an Ad vector, miR-122 sensitized HCC cells to adriamycin and vincristine treatment by causing cell cycle arrest at the G2/M phase and by modulating the expression of multidrug resistant protein, MDR-1 [109]. Using several 3*'* cholesterol-conjugated, 2*'*-O-Me oligonucleotides and an unconjugated 2*'*-O-methoxyethyl-phosphorothioate-modified oligonucleotide, miR-122 was inhibited in mouse livers to demonstrate its function in the lipid metabolism [110,111]. Elmen *et al*. also subsequently reported the inhibition of miR-122 in the mouse liver after the systemic administration of a 16-mer LNA-modified antimiR oligonucleotide [112]. These studies provide strong evidence that miR-122-based HCV therapy can be developed with antagomiRs. To pursue this lead, Lanford *et al*. administered a LNA-modified phosphorothioate oligonucleotide (SPC3649) complementary to the 5*'*-end of miR-122 in chimpanzees with chronic HCV infection and showed that SPC3649 suppression of miR-122 had long-lasting suppression of HCV viraemia with no evidence for viral resistance or side effects in the treated animals [113]. In addition to demonstrating the feasibility and safety of prolonged administration of a LNA oligonucleotide drug *in vivo*, this study showed that miR-122 is essential for HCV accumulation and that the miR-122 seed sites were conserved in all HCV genotypes and subtypes, suggesting the genotype independent nature of this therapy. More recently, Janssen *et al*., further extended this approach and reported successful therapy in HCV patients with an oligonucleotide drug, MiraVirsen, that targets miR-122 [114]. This was the first miRNA-based therapeutic drug developed to treat a liver disease and is currently being tested by Santaris Pharma (Hørsholm, Denmark) in phase 2 clinical trials [28].

These are some of the promising candidates for miRNA replacement therapy for HCC. It is important to exercise caution while designing clinical trials with miRNA mimics for targeting multiple genes relevant to human disease, because of the concerns about potential toxicity in normal tissues, especially under conditions where the therapeutic delivery of miRNA mimics will also lead to an accumulation of exogenous miRNA in normal cells. These toxic effects might be the result of overloading RISC with the exogenous miRNA, thereby competing with endogenous miRNAs necessary for normal cellular welfare and/or hyperactivating cellular pathways that will also reduce the viability of normal cells [46].

An important aspect to consider for miRNA-based therapy is the assessment of off-target effects of miRNA modulators. When using these oligonucleotides, there is a potential risk of affecting cellular RNAs other than the target miRNA [115]. Therefore, proper understanding on the effects of unwanted interactions between antimiR molecules and endogenous RNAs and appropriate designing of antimiRs to minimize the off-target effects is critical. As discussed earlier, the incorporation of chemical modifications, such as LNAs, into antimiRs have been shown to improve mismatch discrimination between a given antimiR and its target miRNA. However, these exogenous miRNAs compete with endogenous ones for miRISC, causing the saturation of miRISC complexes, resulting in the loss of endogenous miRNA regulation and fatality, due to liver toxicity, as demonstrated in animal studies [116,117].

## 5. Perspectives

Because of their functionality in diverse cellular events, miRNA are widely studied in cancer research, as evidenced by hundreds of clinical trials that are currently underway [118]. Moreover, there is a surge in the filing of patent applications worldwide on the use of miRNA in cancer therapeutics [119]. HCC is a multifactorial disease. Therefore, the expression of a large number of genes, proteins and other molecules from diverse cellular processes and pathways are altered in HCC. Hence, the use of a combination therapy that targets multiple different steps and pathways, rather than a single test or a set of tests, might be an appropriate strategy to combat human HCC. miRNAs fit this bill perfectly, as they are capable of targeting many mRNAs simultaneously. Positive therapeutic outcomes achieved with sorafenib that can inhibit receptor tyrosine kinases of multiple signaling cascades, as well as evidence that a single molecule miR-26a can significantly reduce HCC without any toxicity, demonstrate the success of this multi-pronged approach. The main challenge for successful translation of these strategies into the clinic to harness the full potential of miRNAs for therapeutic development remains in vivo delivery. Novel approaches to enhance the stability of miRNA modulators *in vivo* and methods to deliver them efficiently to the target tissues are urgently needed to overcome this challenge.
